# Practical feature filter strategy to machine learning for small datasets in chemistry

**DOI:** 10.1038/s41598-024-71342-1

**Published:** 2024-09-03

**Authors:** Yang Hu, Roland Sandt, Robert Spatschek

**Affiliations:** 1 Institute of Energy Materials and Devices IMD-1, Forschungszentrum Jülich GmbH, 52428 Jülich, Germany; 2 Georesources and Materials Engineering, RWTH Aachen University, 52062 Aachen, Germany; 3 JARA Energy, 52428 Jülich, Germany

**Keywords:** Chemistry, Materials science, Mathematics and computing, Physics

## Abstract

Many potential use cases for machine learning in chemistry and materials science suffer from small dataset sizes, which demands special care for the model design in order to deliver reliable predictions. Hence, feature selection as the key determinant for dataset design is essential here. We propose a practical and efficient feature filter strategy to determine the best input feature candidates. We illustrate this strategy for the prediction of adsorption energies based on a public dataset and sublimation enthalpies using an in-house training dataset. The input of adsorption energies reduces the feature space from 12 dimensions to two and still delivers accurate results. For the sublimation enthalpies, three input configurations are filtered from 14 possible configurations with different dimensions for further productive predictions as being most relevant by using our feature filter strategy. The best extreme gradient boosting regression model possesses a good performance and is evaluated from statistical and theoretical perspectives, reaching a level of accuracy comparable to density functional theory computations and allowing for physical interpretations of the predictions. Overall, the results indicate that the feature filter strategy can help interdisciplinary scientists without rich professional AI knowledge and limited computational resources to establish a reliable small training dataset first, which may make the final machine learning model training easier and more accurate, avoiding time-consuming hyperparameter explorations and improper feature selection.

## Introduction

Machine learning (ML) nowadays plays a central role for many applications in chemistry, physics and materials science due to its potential to unravel unexplored relations and to predict properties, which are hard to access with established experimental or computational methods. However, the required training datasets in several of these disciplines are often rather small and hard to expand due to high experimental efforts and costs^1^. Unfortunately, having sufficiently large and reliable training datasets is an essential precondition for a good model. Therefore, feature selection is considered a key determinant for obtaining an optimal model under these circumstances, and, in turn, a suboptimal feature selection can have a huge detrimental impact on the predictive capabilities of the final model^2^. In general, different algorithms possess distinct sensitivities to the training dataset types and have different advantages in dealing with different problems. A good input feature selection sets the model’s upper limit for the prediction quality, and consequently, different algorithms and their hyperparameters can strongly affect how close the model approaches this accuracy limit^3^. Feature selection is the initial step to build a training dataset, and the initial features guess is normally according to experience. For example, Yin et al. used atomic mass, radius and electronegativity to predict the oxygen vacancy formation energy of perovskite structures using only 110 data points^4^. Zhang et al. use the atomic radius as the central input to predict the lattice constant of $\hbox {A}_{2}\hbox {XY}_{6}$ perovskite structure via just 79 data points^5^. Although deep learning could in general automatically extract useful features from the raw data, the feature engineering for traditional machine learning methods needs to select the input features manually, and this process demands additional efforts and computational costs^6^. We mention that a good feature selection could still improve deep learning’s performance or reduce the number of input features. Moreover, deep learning is not always superior to traditional machine learning algorithms, especially since the required computational resources for traditional machine learning algorithms are usually significantly lower than for deep learning. Especially for small datasets, traditional ML algorithms often possess a better performance than deep learning approaches^7^. However, for manual feature selection, people often prefer to list all possible input features without considering dimensional increases. This can lead to the curse of dimensionality (Hughes phenomenon), i.e., when the number of dimensions or features increases with a training sample with a fixed size, the average predictive power of a classifier or regressor may initially improve. However, beyond a certain point of dimensionality, the predictive power starts deteriorating rather than steadily improving^8–10^. Several scientists have proposed strategies to train a model with a small dataset in physics, chemistry and materials science. For example, Zhang et al. proposed a strategy by incorporating the crude estimation of properties in the feature space to establish ML models using small-sized materials data and succeeded in increasing the prediction accuracy^11^. Vanpoucke et al. have improved the model accuracy by the introduction of an ensemble-averaged model^12^. However, for the feature selection process, a general, simple and practical method for ML practitioners in different interdisciplinary areas is still lacking.

Recently, the Automated Machine Learning (AutoML) technique has appeared, which can automatically train an acceptable model with several algorithms efficiently, as demonstrated in various applications^13^. As an example, Celik et al. predicted the lithium battery cycle lifes by using the $\hbox {H}_{2}\hbox {O}$ AutoML library, which leads to a final accuracy of 99.81% for the cycle life^14,15^. Musigmann et al. also used $\hbox {H}_{2}\hbox {O}$ AutoML for potential applications in diagnostic neuroradiology and it shows promising results^16^. From an application perspective, AutoML may appear as a “black box”, especially for users from a non-machine learning background^17^. Such tools are extremely valuable for generating predictions and unraveling correlations that are difficult to see directly even for small data sets. Still, ideally and manually adjusted models may still be superior to such semi-automatic treatments. The latter allows to focus more on the materials science perspective in the present case. Moreover, several open-source alternatives to AutoML are available like Auto-Sklearn^18^, TPOP2^19^, PyCarret^20^, AutoGluon^21^, and Automatminer^22^, which in principle allow also to understand the workflows in full detail. For the present approach, AutoML’s high efficiency and low threshold concerning required AI skills and computational resources are beneficial.

In the present paper, we use AutoML approaches for an efficient parameter filtering strategy for problems with small training data sets. Next to the approach pursued here, also alternative methods like the aforementioned packages, recursive feature elimination, feature selection using SelectFromModel and L1-based feature selection in the Scikit-Learn^23^ could be used. To make the present approach as transparent as possible, we study two different use cases, namely adsorption and sublimation, which are common and significant processes in chemistry, to highlight the approach. Adsorption describes the attachment of molecules and atoms on a surface and plays an important role in various chemical areas, such as catalysis. The adsorption energy is a significant concept for the analysis of this process, which is often computed via density functional theory (DFT) calculations. Toyao et al. used several ML algorithms to investigate the adsorption of $\hbox {CH}_{4}$ and related species, like $\hbox {CH}_{3}$, $\hbox {CH}_{2}$, CH, C and H on Cu-based alloys and achieved remarkable results^24^. Sublimation plays a central role in a wide range of physical-chemical problems and can e.g. be responsible for various degradation phenomena induced by the evaporation process of Cr-related gaseous species in solid oxide fuel cells (SOFCs)^25^. As long as the sublimation requires only to overcome a single energy barrier, the vapor pressure *p* is described by an Arrhenius-type sublimation function,

$$\begin{aligned} \ln p=-\Delta H_{{\rm sub}}/RT+\ln A. \end{aligned}$$

Here, $\Delta H_{{\rm sub}}$ is the sublimation enthalpy in the sense of the aforementioned energy barrier, and *A* is the prefactor, *R* and *T* are the ideal gas constant and the temperature, respectively. Knudsen effusion mass spectrometry (KEMS) can be used for the experimental determination of the sublimation function-related parameters^26^. For a solid material, the vapor pressure is usually low, especially at low temperatures, which can make its experimental determination difficult. Complementary, computational methods were developed to investigate the sublimation enthalpy, in particular using density functional theory (DFT). We have developed a physical model to predict the sublimation behavior by combining DFT calculations and statistical mechanics, exhibiting a good performance for several compounds^27,28^. However, quantum chemistry methods have high computational demand, and the development of suitable physics-inspired models for the prediction of the vapor pressure through elementary properties of atoms and molecules is required to allow for high throughput material screening computations. During the past years, several researchers have tried to use ML to predict the sublimation enthalpy. Nastaran et al. use quantitative structure-property relationships (QSPR) modeling to predict the enthalpy and Gibbs energy of sublimation, where the most robust and predictive model is constructed by linear regression, which could be improved by neural networks^29^. Sabrina et al. use a Gaussian process regression model with different features, namely E-state fingerprints, custom descriptor set and sum over bonds for organic compounds for the prediction of the sublimation enthalpy^30^.

In the present article, we propose a practical feature filter strategy to build a reliable training dataset for preconditioning the ML model training. To this end, the training dataset of adsorption energies is taken from the literature^24^. According to our filter approach, a reduction of the given 12 features to just a two-dimensional (2D) configuration space becomes possible, which is then used for further ML training. For this second step, 5 different ML algorithms, namely, extra tree regression (ETR), extreme gradient boosting regression (XGBoost), support vector regression (SVR), decision tree regression (DTR) and Gaussian process regression (GPR) deliver a higher accuracy, compared to reported values using models with higher dimensional input in the literature. For the sublimation enthalpies prediction, we use data from the thermodynamic databases in FactSage^31^. In total, not more than 177 different pure substances, each containing a maximum of two types of elements, are chosen for model training. Furthermore, eight additional substances including Sr, Ni, Cu, Cr, NaCl, NaF, $\hbox {SiO}_{2}$ and $\hbox {ZrO}_{2}$ constitute our prediction dataset. Here, we apply the filter strategy with the help of AutoML for screening suitable and relevant feature combinations from 14 different configurations consisting of 8 initial input feature candidates. These basic input feature candidates are considered based on general physical arguments and expectations. Several possible input candidate groups are investigated in AutoML as a prescreening step, and the final input candidate set is selected from the minimization of the average mean absolute error ($\overline{\text {MAE}}$) and used for further refined ML simulations. As for the adsorption example, we train our ML models with four different algorithms, namely, XGBoost, SVR, DTR and GPR as a second step for obtaining a refined model. The evaluation of the models’ accuracies is carried out from two perspectives: First, a statistical evaluation via mean absolute error (MAE) and root mean squared error (RMSE) calculations, and second, a theoretical perspective with the help of the relative feature importance and the Shapley additive explanations (SHAP) value. This analysis makes our ML model interpretable and illustrates the contribution of each input feature to the final prediction directly, allowing us to understand the features’ relevance and supporting a physical understanding, which confirms the accuracy and their theoretical background very well. The manual hyperparameters optimization process is with the help of the GridsearchCV optimization algorithm in the different ML methods. The predicted sublimation enthalpies of the prediction dataset are compared to the initial FactSage training dataset and independent DFT calculations.

## Results

### Feature filter process using AutoML

**Figure 1:**
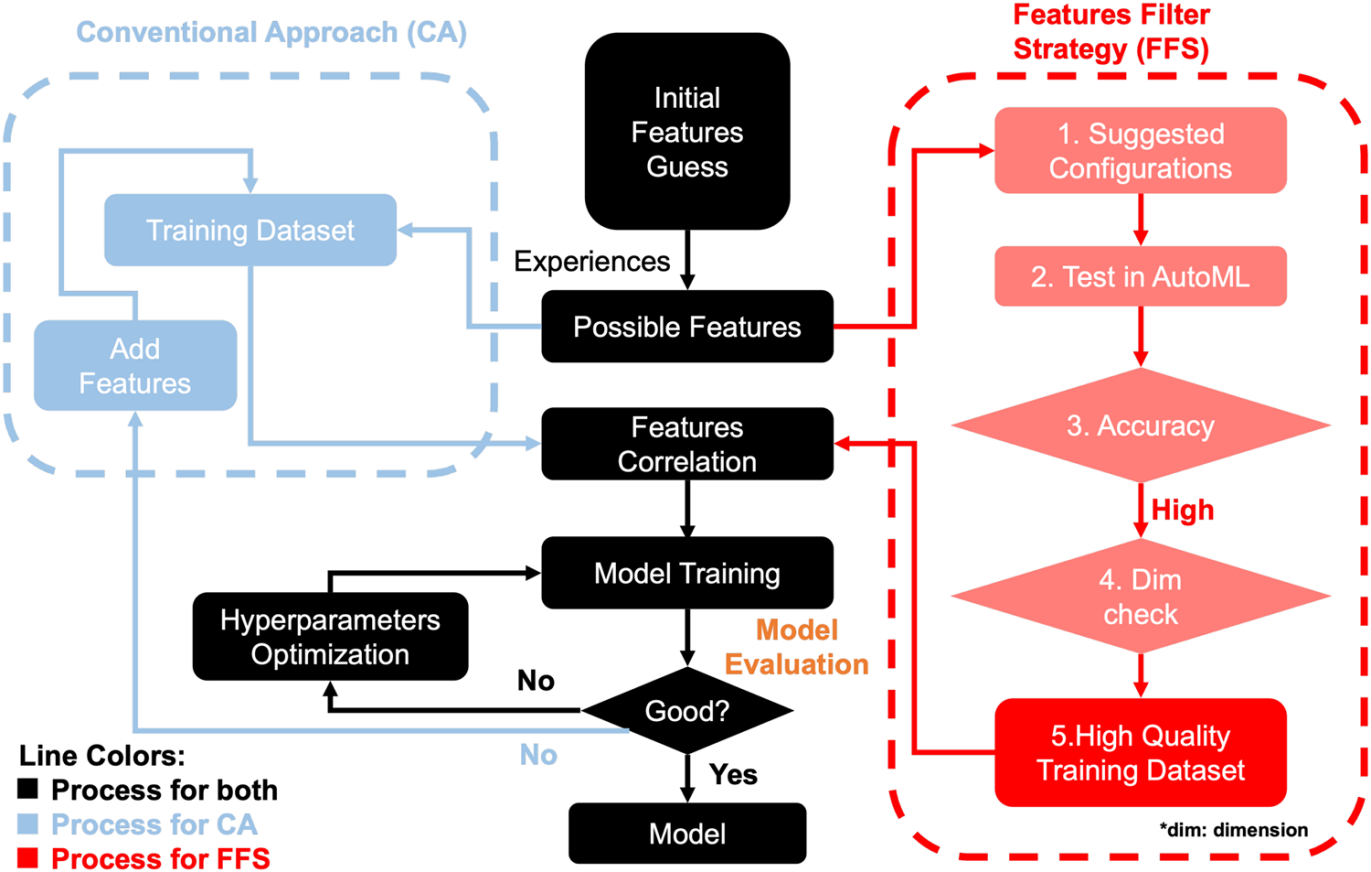
Illustration of the computational workflow with feature filter strategy. The black part of the diagram is common to both the conventional feature selection route and the novel approach. The blue parts are specific to the manual selection in the conventional approach, whereas the red route illustrates the workflow for the new methodology.

The conventional approach to select features is illustrated by the black and blue parts of the flow diagram in Fig. 1, as compared to the novel approach which uses the red instead of the blue branch for the feature selection. In the conventional approach, after the initial guess of features, which is typically according to personal experience, the possible features are directly used to build the training dataset. When the model’s performance is not as expected, the hyperparameters are optimized and more features may be considered in the training dataset. However, this process can be long-lasting and may require substantial manual work, and typically depends strongly on personal expectations and biases. A further improvement of the accuracy by optimization of the hyperparameters could be very hard, and adding new features is risky as it may lead to the curse of dimensions.

In contrast, the newly proposed approach (red branch) uses an initial filter strategy to select the suitable features first, instead of directly using all feature candidates, as this latter approach could lead to serious drawbacks from the high dimensional configuration space. Therefore, the AutoML approach can test many different algorithms with high computational efficiency simultaneously to obtain an acceptable initial ML model, and each test typically takes only a very short time (for the chosen examples, only around 5 seconds per test), depending on the specific problem and settings. After the initial feature guess, the suggested configurations are listed and imported into AutoML for a quick test. The suggested configurations are selected based on physical and chemical reasoning. In the conventional approach, all possible features are listed, however, some features may not have extremely high correlations. In contrast, in the present process, we aim to use our strategy to filter these less relevant features. At this stage, the final training dataset candidate has to satisfy two main standards: (i) relatively higher accuracy than the other candidates; (ii) lower dimensions with comparable accuracy. Finally, a high-quality training dataset is utilized for the model training and further model improvements can be restricted to a hyperparameter optimization. Overall, this strategy possesses three main advantages, especially for small datasets: First, it avoids the curse of dimension, second, it avoids a bias in the initial feature guesses, third, it saves time for the hyperparameter search by skipping reasonless feature selection steps, and fourth, it provides an initial accuracy benchmark for further model training.

In the following, all necessary steps, which are given in the flow diagram in Fig. 1, are illustrated in detail for the two examples of the adsorption and sublimation enthalpies.

### Adsorption energy

We base the analysis for the adsorption energy prediction on the careful analysis and data provided by Toyao et al.^24^. They have used 4 different ML algorithms to predict the DFT calculated adsorption energies of $\hbox {CH}_{4}$ related species, like $\hbox {CH}_{3}$, $\hbox {CH}_{2}$, CH, C and H on Cu-based alloys depending on the 46 data points training dataset by using 12 features, the train/test split ratio is fixed as 0.75 first, the evaluation process is a statistical result via calculation of the average RMSE ($\overline{\text {RMSE}}$) of different test datasets for 100 times. Here, we used only the prediction of adsorption energy of $\hbox {CH}_{3}$ as an example. We first performed univariate analyses using Kendall’s tau coefficient (KTC), which is suitable for a small database. It shows that $T_m$ and *SUE* have a higher KTC, and *SUE* has the highest KTC. However, other parameters do not show clear correlations (Supplementary Fig. 1), therefore suggesting to use further machine-learning approaches. As algorithms, ordinary linear regression by least squares (OLR), random forest regression (RFR), gradient boosting regression (GBR) and ETR were employed. After that, other 10D, 6D, 5D and 3D input feature sets were tested, chosen according to the relative feature importance, as obtained through ETR^24^.

**Figure 2:**
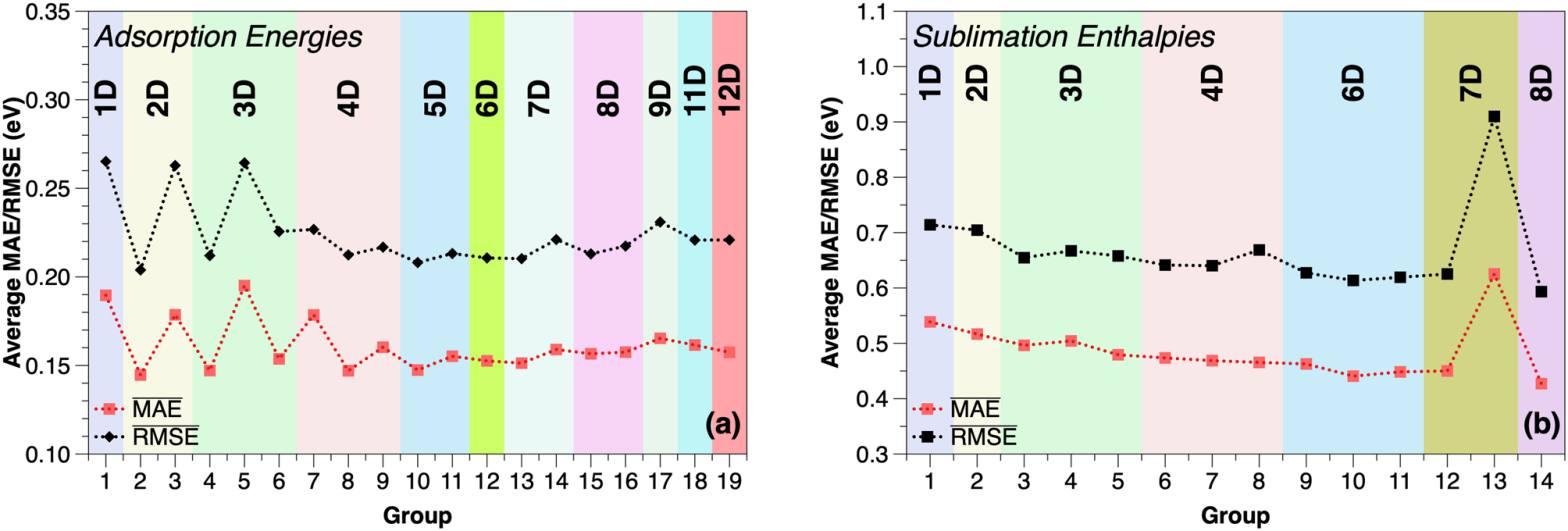
Comparison of accuracy in prediction of and (**a**) adsorption energies and (**b**) sublimation enthalpies based on the AutoML tests for different feature groups. The continuous curves are a guide to the eye only.

In the current paper, 12 initial features are employed for the filtering process, as suggested by the authors of the previous study^24^. These are the atomic number in periodic table *AN*, atomic mass *AM*, group *G*, period *P*, atomic radius *R*, electronegativity $\chi$, melting temperature $T_m$, boiling temperature $T_B$, enthalpy of fusion $\Delta H_{fus}$, density $\rho$, ionization energy *IE* and surface energy *SUE*. For the filtering process, we follow the red route in Fig. 1. As the first step, 17 different configurations are employed for this part (Supplementary Table 1), to identify reasonable parameter combinations. The determination of the configuration considers the similarity of physical properties, such as the $T_m$, $T_B$ and $\Delta H_{fus}$. In the second step, these 17 configurations are quickly tested in AutoML. To be comparable to the previous work^24^, we keep the same split ratio of 0.75. We concentrate on the average errors of all predictions, hence $\overline{\text {MAE}}$ is used as an error metric. Since RMSE is utilized as the evaluation criterion in the proceeding analysis, the related RMSEs are also recorded here (Supplementary Table 1 and Table 2). Figure 2a shows the $\overline{\text {MAE}}$ and $\overline{\text {RMSE}}$ of the prescreening tests (steps 3 & 4).

Overall, the accuracy fluctuates in a certain range and stabilizes beyond a feature space dimension of four. Furthermore, as the dimensionality increases, $\overline{\text {MAE}}$ and $\overline{\text {RMSE}}$ exhibit a slight upward trend. This trend is more pronounced in terms of $\overline{\text {RMSE}}$ beyond 7D, which may indicate the Hughes phenomenon. Finally, as the results of the fifth prescreening step, we consider the two-dimensional group 2 with high accuracy but with the lowest dimension for further exploration.

**Figure 3:**
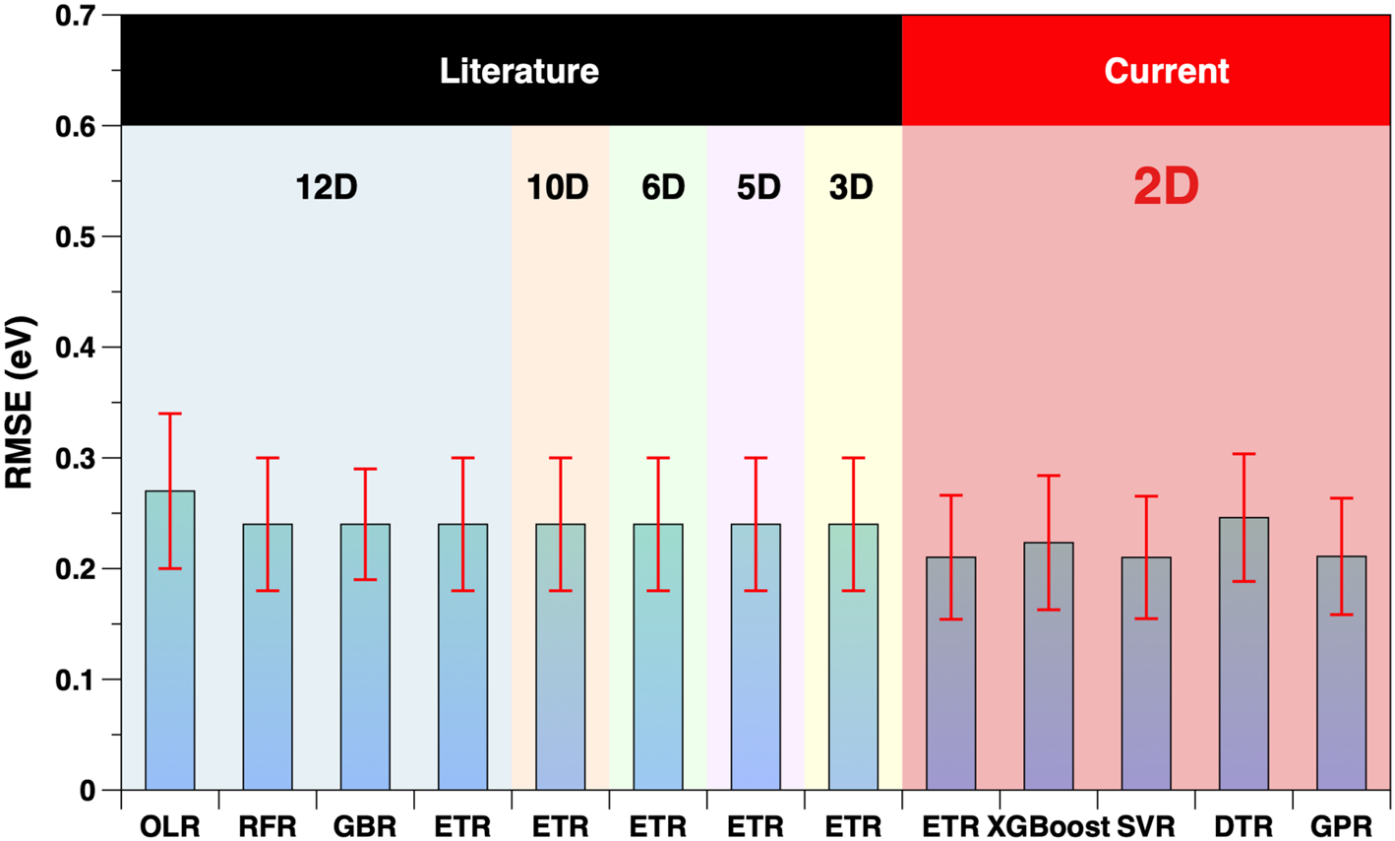
Comparing the $\overline{\text {RMSE}}$ (eV) of 2D input for adsorption energies to that of the 3-12 dimensional input in the literature^24^, using 100 tests.

To possibly improve these predictions, we use XGBoost, SVR, DTR and GPR algorithms for the model training. As ETR was used in the preceding analysis^24^, we additionally employ this approach here. The dataset is split with the split ratio of 0.75 as before, and each model is tested 100 times, for better comparability to the reference work^24^. Therefore, 100 random test datasets were created (Supplementary Table 1 and Table 4). Figure 3 compares the $\overline{\text {RMSE}}$ of the five different ML models by using the previously selected 2D input to that of the 8 previously reported different models with 3-12 dimensional input feature spaces^24^.

Overall, all models exhibit similar performance, and the 2D model based on the previously filtered features shows a slight accuracy advantage, which is similar to the RMSE as obtained through the AutoML prescreening only. This example shows that the parameter filter strategy has effectively reduced the dimensions from 12D to 2D, while maintaining accuracy. As the data set contains only 46 data points, this drastically reduces the sparsity in the configuration space and therefore allows for more robust predictions.

### Sublimation enthalpy

For the example of sublimation enthalpies, eight different features are used as feature candidates according to general experience. In detail, we use the atomic number of the (gaseous) molecules (*N*), their atomic radius (*R*), atomic mass (*m*) and electronegativity ($\mathrm \chi$) and melting temperature ($T_m$), as they are expected to influence the chemical bond strength. Most of these variables exist for both A and B elements in compounds of the type $\hbox {A}_{\textrm{n}}\hbox {B}_{\textrm{m}}$, hence the total variable set contains the 8 independent features *N*, $R_A$, $R_B$, $m_A$, $m_B$, $\chi _A$, $\chi _B$ and $T_m$. Similarly to above, a univariate analysis is employed first, and it shows clear correlations between the melting point and the sublimation enthalpy, which is plausible from physical arguments, but is not conclusive concerning the other feature variables (Supplementary Fig. 2). Therefore, from the above variable set, we constitute 14 different configurations(step 1) (Supplementary Table 5). It should be mentioned that several of these combinations may lack practical relevance and are only used for comparison and demonstration of the general strategy. In the second step, we employ again AutoML for the test of different ML models. The $\overline{\text {MAE}}$/$\overline{\text {RMSE}}$, as obtained from different random training datasets by using the common split ratio of 0.8, is then used as an error metric (Supplementary Table 5 and Table 6). Figure 2b compares the $\overline{\text {MAEs}}$/$\overline{\text {RMSEs}}$, showing that the model’s accuracy generally improves with an increase in input feature dimension and finally stabilizes beyond the six-dimensional (6D) input set. One 7D outlier without the input feature $T_m$ possesses an extremely high error, indicating the importance of the melting point to the final prediction (steps 3 &4). Considering the comparable high accuracy of group 14 (8D) and the comparable performance of group 7 (6D), these 2 groups are allowed to be the candidates for the next ML training round (step 5). For the explanation of the dimensional effect, group 12 (7D) is also taken into account for this next round.

**Figure 4:**
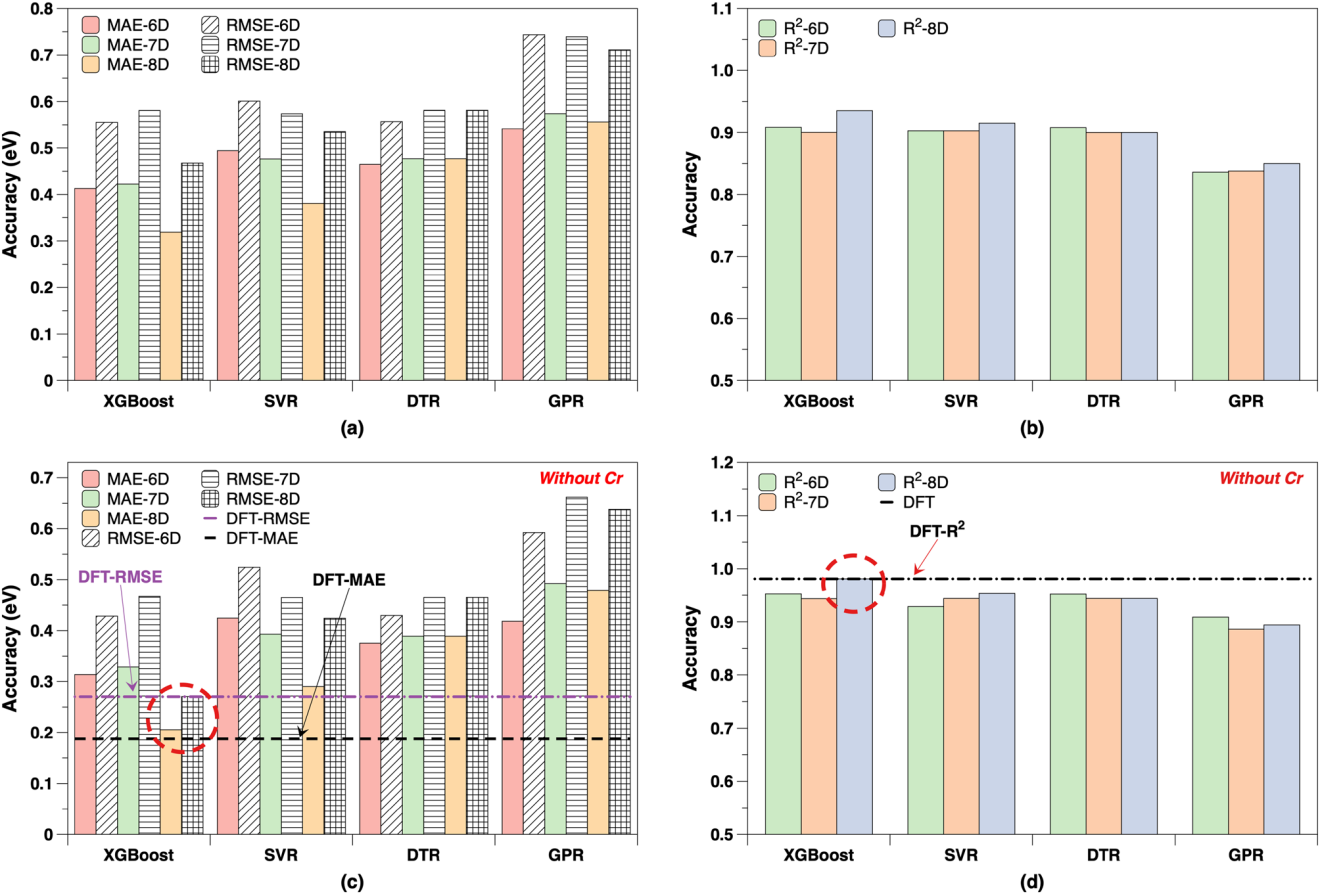
Comparison of sublimation enthalpy results of the (**a**) MAE and RMSE, (**b**) $\hbox {R}^2$ of four different ML algorithms with 6D-8D inputs; (**c**) $\hbox {MAE}_{\textrm{w}}$, $\hbox {RMSE}_{\textrm{w}}$, (d) $\hbox {R}^2_w$ are that without outlier Cr. The hists in (**a**,**c**) with solid colors and the pattern are the MAE and RMSE, respectively; the dashed lines in (**c**,**d**) are the previous DFT calculations.

Based on the above preselection of features, we develop ML models to predict sublimation enthalpies, using MAE, RMSE and R-Squared ($\hbox {R}^2$) as error metrics. The ML models are developed with four different algorithms, which have been used to predict eight untrained substances (prediction dataset). All results are listed in (Supplementary Table 7). Figure 4a compares the MAE and RMSE of the four different ML algorithms with three different input dimensions (6D, 7D and 8D), which are considered as the competitive input candidates, as elaborated above.

The RMSE is larger, as it is more sensitive to large errors and outliers than the MAE. The comparison of the $\hbox {R}^2$ is shown in Fig. 4b, and the closer $\hbox {R}^2$ gets to 1, the better the result. In general, all models capture the general trends of the AutoML tests well but with higher accuracy. With the exception of the GPR model, the models possess a good performance, and XGBoost, trained with eight features (8D-XGBoost), gives the highest accuracy with a MAE of 0.3189 eV, RMSE of 0.4675 eV and a highest $\hbox {R}^2$ of 0.9325. The SVR model performs better as the dimensionality increases, such as the MAE decreased from 0.4940 eV in six dimensions to 0.3805 eV in eight dimensions. The DTR models exhibit a relatively similar performance across all dimensions.

One outlier (Cr) is observed in the plot of the predicted values versus the true values (Supplementary Fig. 3), thus, we calculate also the corresponding MAE, RMSE and $\hbox {R}^2$ without Cr, namely, $\hbox {MAE}_{\textrm{w}}$, $\hbox {RMSE}_{\textrm{w}}$ and $\hbox {R}^2_{\textrm{w}}$. Figure 4c,d compare the $\hbox {MAE}_{\textrm{w}}$, $\hbox {RMSE}_{\textrm{w}}$ and $\hbox {R}^2_{\textrm{w}}$ of our models to DFT results. Here, the purple and black dashed line in Fig. 4c represents the $\hbox {MAE}_{\textrm{w}}$, $\hbox {RMSE}_{\textrm{w}}$ of the DFT calculations, and the black dashed line in Fig. 4d is the $\hbox {R}^2$ value. The red dashed circles in both figures compare the results of the 8D input feature XGBoost model to the DFT results. It is worthwhile to mention that the 8D XGBoost reaches almost the same accuracy level as the independent DFT calculations. Hence, by excluding the outlier Cr the improvement in the accuracy of all models becomes clearly evident. It should be noted that the ML models have a significantly lower computational effort, as compared to the quantum mechanical calculations with the required structure optimizations of the solid and gaseous phases, and the new approach could even be improved by the expansion of the training dataset.

**Figure 5:**
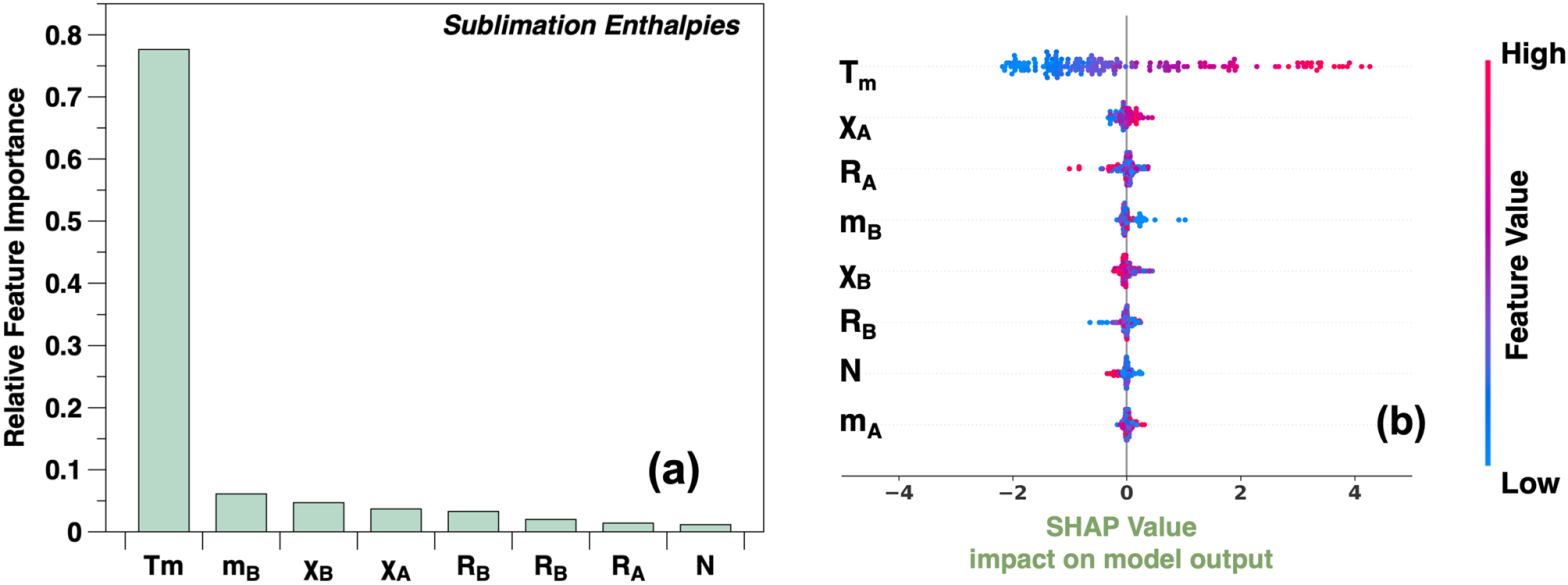
(**a**) Relative feature importance and (**b**) SHAP values of the 8D-XGBoost model for the sublimation enthalpies.

Apart from the evaluation from a statistical perspective, we also explore physical and chemical interpretations of the trained models, which is independent of the above feature selection. The final model (8D-XGBoost) is an interpretable tree-based XGBoost algorithm. The theoretical explanation of the predictions is carried out with the help of the relative feature importance and SHAP value calculations, as shown in Fig. 5.

According to the relative feature importance in Fig. 5a, the melting temperature $T_m$ has a dominant influence with the highest contribution of 77.63%. This is in agreement with the physical expectation that a higher melting point $T_m$ reflects a higher bond strength inducing a higher sublimation enthalpy. The atomic mass of the B-side element $m_B$ takes second place with a contribution of only 6.11%, followed by the electronegativity of the B-side element (4.71%). Figure 5b indicates the results of SHAP value calculations, where the color reflects the feature values versus SHAP values. A positive (negative) SHAP value indicates a positive (negative) impact of this data point on the prediction, and a larger absolute SHAP value indicates that the influence is higher. Here, we focus only on the top three features in contribution analysis. For the melting temperature $T_m$, the impact thereof on the final prediction is almost linear, and a higher melting temperature $T_m$ has a more positive influence on the final prediction. For the atomic mass of the B-side element $m_B$, some data points with very low mass $m_B$ have a positive impact on the prediction. For the outlier Cr the relation between sublimation enthalpy and melting temperature is inverted, if compared e.g. to Fe. In this case, inclusion of additional features like the boiling temperature could lead to improved predictions.

**Figure 6:**
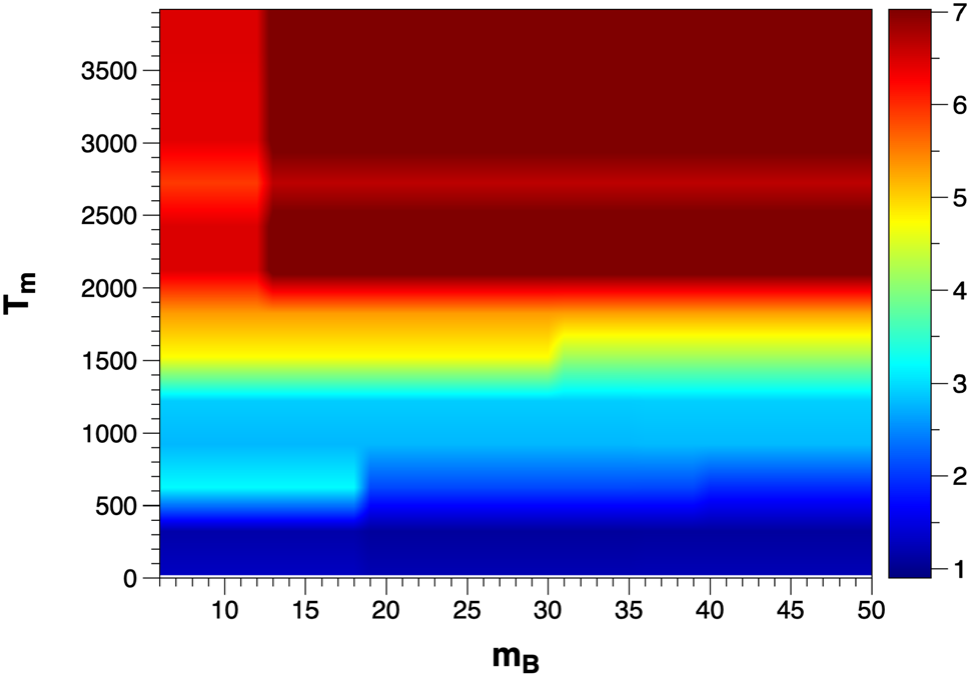
Heatmap of the predicted sublimation enthalpy versus $T_m$ and $m_B$ by using the 8D-XGBoost model; the features $N=2$, $r_A=100$ pm, $r_B=130$ pm, $m_A=130$ u, $\chi _A=1$ and $\chi _B=1$ are kept fixed.

For a physical and chemical interpretation and prediction of the sublimation enthalpy, a dimensional reduction from the originally eight-dimensional space based on the observed main dependencies is beneficial. Therefore, we use a heatmap plot in Fig. 6 for the predicted sublimation enthalpy as a function of the dominant features $T_m$ and $m_B$, assuming that all other, less relevant features are kept constant.

The figure shows a similar trend as the above relative feature importance and SHAP analysis, and again a higher melting temperature $T_m$ leads to a higher sublimation enthalpy. Nevertheless, in certain specific parameter ranges with a lower mass $m_B<30\textrm{u}$ a higher sublimation enthalpy is predicted than by the melting temperature alone. In the periodic table, B site elements with small mass and compounds with high melting temperature $T_m$ are quite common, like B, C and N, forming borides, carbides and nitrides. Such compounds typically exhibit elevated levels of hardness and melting points, hence also high sublimation enthalpies can be expected. It is worthwhile to mention that the training dataset does not include borides and carbides. Nitrides are also expected to have high sublimation enthalpies due to the low mass of nitrogen, and indeed AlN with a sublimation enthalpy of 7.58 eV is contained in the training dataset. We can therefore conclude that the models lead to good predictions of the sublimation enthalpy despite the small training dataset, and the predictions are in line with fundamental physical and chemical expectations and arguments.

## Discussion

In this paper, we have proposed a practical feature filter strategy for ML applications e.g. in materials science, physics and chemistry, where often only small training data sets are available. As a first example, we have used an open training dataset concerning adsorption energies of $\hbox {CH}_{4}$ related species $\hbox {CH}_{3}$ on Cu-based alloys. Using the proposed approach, we succeeded in reducing the initial 12-dimensional feature space to just 2D with even slightly increased accuracy. This reduction of features is obtained through a simple and computationally inexpensive approach, reducing not only the risk of the curse of dimensions but also allowing for an easier physical interpretation of the predictions. The same approach is also demonstrated using an example of sublimation enthalpies prediction for binary compounds, based on a small dataset. The filter approach identifies the melting temperature of the compound and the mass of the B site element as the most important feature variables, which are in agreement with general physical arguments and experimental experience e.g. on borides, carbides and nitrides. We believe that similar results can be obtained for other applications, where otherwise the excessive use of ML techniques is hampered by small training data set sizes. From a practitioner’s perspective it is often not required to determine the proven optimum algorithm, but rather an easy to use approach like the suggested one here, which is able to combine statistical and data driven approaches with physical background knowledge.

The proposed parameter filter strategy reduces not only the dependence of feature selection on personal experience and expectations but also accelerates this process significantly through the use of AutoML approaches. Moreover, the convolutional process often requires high computing efforts and also high personal AI skills, which is usually hard for interdisciplinary scientific work. The construction of a training dataset is an essential precondition to obtaining a good model, and the challenge of a small database must not be ignored, as then a suitable feature selection is even more critical. Therefore, the suggested feature filter strategy can be helpful, particularly for scientists without rich professional artificial intelligence knowledge and enough computational resources to obtain a reliable small dataset practically and quickly, which could also avoid the curse of dimensions, reducing computing time and setting a solid foundation for further model training steps and interpretation of the results.

## Methods

### Data preparation

**Adsorption Energies.** The dataset for Adsorption Energies prediction is taken from the literature^24^, which contains 46 data points. 12 initial features are employed, namely atomic number in the periodic table *AN*, atomic mass *AM*, group *G*, period *P*, atomic radius *R*, electronegativity $\chi$, melting temperature $T_m$, boiling temperature $T_B$, enthalpy of fusion $\Delta H_{fus}$, density $\rho$, ionization energy *IE* and surface energy *SUE*. The label is the DFT calculated adsorption energies of $\hbox {CH}_{4}$ related species $\hbox {CH}_{3}$ on the Cu-based alloys.

**Sublimation Enthalpies.** The training dataset contains in total of 177 different pure substances, and an additional prediction dataset including 8 different pure substances is built using the FactSage database (mainly FactPS, SGPS)^31^. The prediction database consists of Sr, Ni, Cu, Cr, NaCl, NaF, $\hbox {SiO}_{2}$ and $\hbox {ZrO}_{2}$, which are also the calculated examples via DFT^27,28^. These selected pure substances differ by bond properties, namely, metallic bond, ionic bond and covalent bond, therefore representing a broad range of compounds as well as molecular structures in the gas phase. We consider vaporization according to the reaction

1 $$\begin{aligned} \mathrm A_{m}B_{n}(s)\rightarrow {\textrm{A}}_{m}B_{n}(g), \end{aligned}$$

without dissociation of the molecules.

After the data extraction from the database, the initial input features are determined using prior scientific experience. The analysis and calculations in the aforementioned earlier publications show that the sublimation enthalpy is directly related to the bond energy. Therefore, we first propose several basic physical properties, which may contribute to the bond strength and also help to distinguish the atom types. Therefore, the feature candidates are the atomic radius (*r*), the atomic mass (*m*) and the atomic electronegativity ($\chi$). In detail, we use the calculated atomic radii by Clementi et al.^32^; the atomic mass is from the National Institute of Standards and Technology (NIST) database 78^33^, and the electronegativity is expressed on the Pauling scale^34^. For a binary system, two formula units may contain the same elements but with different atomic numbers, such as $\hbox {WCl}_{4}$ and $\hbox {WCl}_{5}$. The number of atoms in the molecule (*N*) is therefore a further suggested feature. Furthermore, the melting point $T_m$ as a significant high-temperature physical property is also taken into account for polyatomic molecules. For example, for a binary system $\hbox {A}_{n}\,\hbox {B}_{m}$, the A site is considered to be the positive valence site. Molecules consisting of a single atom type are treated as $\hbox {A}_{n}\,\hbox {A}_{m}$, hence the B position is occupied by the same element as the A position. Then, the possible features are combined mathematically and imported into the $\hbox {H}_{2}\hbox {O}$ AutoML for the filtering process. Finally, we have 8 different possible input features: N, $R_A$, $R_B$, $m_A$, $m_B$, $\chi _A$, $\chi _B$ and $T_m$.

### AutoML

The AutoML technique has the ambition to make ML methods more accessible and efficient. Here, $\hbox {H}_{2}\hbox {O}$ AutoML is employed, which is a fully automated algorithm to investigate different ML methods^14^. All tested models are evaluated by different metrics and therefore the algorithm allows an efficient determination of the best approach^35^. To ensure the model’s generalization ability, we use 17/14 different configurations as input candidates for the adsorption energies/sublimation enthalpies, respectively (Supplementary Table 1 and Table 5). AutoML is limited to use 10 different algorithms for both examples. For the adsorption energies prediction, each configuration is tested three times with a train/test split ratio of 0.75 and randomly selected training data sets. The selection of the final input feature combination is made according to the $\overline{\text {MAE}}$ and $\overline{\text {RMSE}}$. For the sublimation enthalpies prediction, we use the common train/test split ratio of 0.8 and three randomly selected training data sets, and $\overline{\text {MAE}}$ is used as the evaluation criterion.

### Machine learning

We use the open-source package Scikit-Learn for all ML methods^23^, namely, ETR, XGBoost, SVR, DTR and GPR. The min-max normalization is employed in the data processing part, which is a common normalization method and has demonstrated advantages for high dimensional data spaces like the sublimation enthalpy example^36^. The manual hyperparameter exploration is done with the help of GridSearchCV from the scikit-learn library and with different metrics (MAE, RMSE and $\hbox {R}^2$) with 3- and 5-fold cross-validation for the sublimation enthalpy data set. For the adsorption enthalpy prediction, we instead use the same test-test split ratio as in Ref.^24^ for better comparability. .

### Shapley additive explanations (SHAP)

The SHAP (Shapley additive explanations) method is a widely used technique in the field of explainable artificial intelligence (XAI), that is grounded on cooperative game theory and offers desirable properties, like interpreting predictions from tree ensemble methods^37,38^. A simpler explanation model is required to better understand the contributions of each input to the final prediction made by an ML model, which is defined as any interpretable approximation of the original model. The final impact of each input on the prediction can be analyzed according to the calculation of the SHAP value, where a positive (negative) SHAP value indicates a corresponding positive (negative) influence on the model output^37,39^.

## Supplementary Information

Supplementary Information.

## Data Availability

The datasets analyzed for adsorption energies are available in the published paper https://pubs.acs.org/doi/10.1021/acs.jpcc.7b12670. The developed codes are available from the public repository (https://github.com/yanghuphysics/Practical-feature-filter-strategy-to-machine-learning-for-small-datasets-in-chemistry.git).
