# Supplementary material for: Practical feature filter strategy to machine learning for small datasets in chemistry
Source: Sci Rep. 2024 Sep 3;14:20449. doi: 10.1038/s41598-024-71342-1 (PMC11379859; doi:10.1038/s41598-024-71342-1)
Supplement: Supplementary file 1 — Supplementary Information. [file 41598_2024_71342_MOESM1_ESM.pdf]

Supplementary Information

Supplementary Table 1. Comparison of the MAE for different input features for the prediction of adsorption energies with AutoML with corresponding best-performing algorithms for each feature configuration. Here, the columns labeled as 1st, 2nd and 3rd have the same split ratio of 0.75 as but use different random seeds to get different training datasets. The averaged MAE ($\overline{\text{MAE}}$) is then used as an error metric.

Group	Features	Algorithms	MAE (eV)			
			1st	2nd	3rd	$\overline{\text{MAE}}$
1	SUE	GLM, GBM, XGBoost	0.1930	0.1933	0.1824	0.1896
2	G, SUE	SE, GBM	0.1616	0.1395	0.1327	0.1446
3	AN, SUE	SE, DRF	0.1937	0.1755	0.1665	0.1786
4	AN, G, SUE	DRF, XRT, GBM	0.1557	0.1432	0.1425	0.1471
5	AN, IE, SUE	GBM, SE	0.2010	0.1938	0.1906	0.1951
6	G, Tm, SUE	GBM, XGBoost	0.1674	0.1514	0.1422	0.1537
7	AN, G, χ, IE	GBM, XRT	0.1898	0.1717	0.1741	0.1785
8	AN, G, IE, SUE	GBM, XGBoot, GLM	0.1598	0.1442	0.1371	0.1470
9	AN, G, χ, SUE	XGBoost, GBM	0.1775	0.1466	0.1568	0.1603
10	AN, G, P, IE, SUE	GBM, XGBoost, SE	0.1574	0.1465	0.1384	0.1474
11	AN, G, P, χ, SUE	GBM	0.1580	0.1451	0.1625	0.1552
12	AN, G, P, χ, IE, SUE	XRT, XGBoost, GBM	0.1613	0.1510	0.1454	0.1526
13	AN, G, P, χ, ρ, IE, SUE	GBM	0.1581	0.1515	0.1442	0.1513
14	AN, G, P, R, χ, ρ, IE, SUE	GBM, XGBoost	0.1635	0.1511	0.1551	0.1566
15	AN, AM, G, P, R, χ, ρ, IE, SUE	GBM, XGBoost	0.1618	0.1513	0.1593	0.1575
16	AN, G, P, R, χ, T_m, T_B, ΔH_{fus}, ρ, IE, SUE	XGBoost, GBM	0.1662	0.1542	0.1642	0.1615
17	AN, AM, G, P, R, χ, T_m, T_B, ΔH_{fus}, ρ, IE, SUE	XGBoost, GBM	0.1605	0.1477	0.1639	0.1574

*__DRF:__ Distributed Random Forest, **XRT:** Extremely Randomized Trees.

Supplementary Table 2. Comparison of the RMSE for different input features for the prediction of adsorption energies with AutoML with corresponding best-performing algorithms for each feature configuration. Here, the columns labeled as 1st, 2nd and 3rd have the same split ratio of 0.75 but use different random seeds to get different training datasets. The averaged RMSE ($\overline{\text{RMSE}}$) is then used as an error metric.

Group	Features	Algorithms	RMSE (eV)			
			1st	2nd	3rd	$\overline{\text{RMSE}}$
1	SUE	GLM, XGBoost	0.2687	0.2666	0.2603	0.2652
2	G, SUE	GBM, XGBoost	0.2264	0.1949	0.1905	0.2039
3	AN, SUE	SE, XGBoost, GBM	0.2783	0.2721	0.2383	0.2629
4	AN, G, SUE	GBM, SE	0.2185	0.2045	0.213	0.2120
5	AN, IE, SUE	GBM, GLM, XRT	0.2716	0.2699	0.2517	0.2644
6	G, Tm, SUE	GBM,XGBoost	0.2365	0.2231	0.2173	0.2256
7	AN, G, χ, IE	GBM, DRF	0.2458	0.2151	0.2195	0.2268
8	AN, G, IE, SUE	XRT, GBM, SE	0.2245	0.2102	0.2025	0.2124
9	AN, G, χ, SUE	GBM, XGBoost	0.2381	0.2094	0.2028	0.2168
10	AN, G, P, IE, SUE	GLM, GBM, SE	0.2158	0.21332	0.1953	0.2081
11	AN, G, P, χ, SUE	GLM, GBM, SE	0.2115	0.2056	0.2225	0.2132
12	AN, G, P, χ, IE, SUE	SE, DRF	0.2256	0.2107	0.1958	0.2107
13	$AN, G, P, \chi, \rho, IE, SUE$	GLM, GBM, SE	0.2077	0.2164	0.2068	0.2103
14	$AN, G, P, R, \chi, \rho, IE, SUE$	GLM, GBM, XGBoost	0.216	0.2147	0.2084	0.2130
15	$AN, AM, G, P, R, \chi, \rho, IE, SUE$	GLM, GBM	0.2129	0.2188	0.2204	0.2174
16	$AN, G, P, R, \chi, T_m, T_B, \Delta H_{fus}, \rho, IE, SUE$	GLM, GBM, SE	0.2061	0.2194	0.2368	0.2208
17	$AN, AM, G, P, R, \chi, T_m, T_B, \Delta H_{fus}, \rho, IE, SUE$	SE, XGBoost	0.2165	0.2218	0.2243	0.2209

Supplementary Table 3. The $\overline{\text{RMSE}}$ of 100 tests for prediction of adsorption energies of CH_4 related species CH_3 on the Cu-based alloys with 12D features in literature[1]

Algorithms	OLR	RFR	GBR	ETR
$\overline{\text{RMSE}}$	0.27	0.24	0.24	0.24
Standard Deviation (eV)	0.07	0.06	0.05	0.06

Supplementary Table 4. Ten tests for the prediction of adsorption energies in (eV), each test takes the split ratio of 0.75 but with different training/test datasets, the random seeds are from 1 to 100.

Metrics	ETR	XGBoost	SVR	DTR	GPR
$\overline{\text{RMSE (eV)}}$	0.2102	0.2234	0.2101	0.2460	0.2110
Standard Deviation	0.056	0.061	0.055	0.058	0.053

Supplementary Table 5. Comparison of the MAE for different input features for the prediction of sublimation enthalpies with AutoML with corresponding best-performing algorithms for each feature configuration. Here, the columns labeled as 1st, 2nd and 3rd have the same split ratio of 0.8 but use different random seeds to get different training datasets, i.e., the data sequence has been shuffled. The averaged MAE ($\overline{\text{MAE}}$) is then used as an error metric.

Group	Features	Algorithms	MAE (eV)			
			1st	2nd	3rd	$\overline{\text{MAE}}$
1	T_m	GLM, SE, GBM	0.5630	0.4985	0.5545	0.5387
2	N, T_m	SE	0.5157	0.4921	0.5419	0.5166
3	R_A, R_B, T_m	SE	0.5364	0.4656	0.4869	0.4963
4	m_A, m_B, T_m	SE	0.5347	0.4594	0.5188	0.5043
5	χ_A, χ_B, T_m	GBM, SE	0.5004	0.4477	0.4899	0.4793
6	N, R_A, R_B, T_m	SE	0.4803	0.4570	0.4835	0.4736
7	N, m_A, m_B, T_m	GBM, SE	0.4647	0.4470	0.4943	0.4687
8	N, χ_A, χ_B, T_m	GBM, SE	0.4346	0.4744	0.4875	0.4655
9	$N, R_A, R_B, m_A, m_B, T_m$	SE	0.4563	0.4427	0.4890	0.4627
10	$N, R_A, R_B, \chi_A, \chi_B, T_m$	SE, GBM	0.4650	0.4291	0.4279	0.4407
11	$N, m_A, m_B, \chi_A, \chi_B, T_m$	GBM, SE, XGBoost	0.4335	0.4306	0.4810	0.4484
12	$R_A, R_B, m_A, m_B, \chi_A, \chi_B, T_m$	GBM, SE	0.4653	0.4357	0.4492	0.4501
13	$N, R_A, R_B, m_A, m_B, \chi_A, \chi_B$	GBM, SE	0.6434	0.6136	0.6191	0.6254
14	$N, R_A, R_B, m_A, m_B, \chi_A, \chi_B, T_m$	GBM, SE	0.4243	0.4156	0.4415	0.4271

*GLM: Generalized Linear Models, **SE:** Stacked Ensemble, **GBM:** Gradient Boosting, **XGBoost:** Extreme Gradient Boosting.

Supplementary Table 6. Comparison of the RMSE for different input features for the prediction of sublimation enthalpies with AutoML with corresponding best-performing algorithms for each feature configuration. Here, the columns labeled as 1st, 2nd and 3rd have the same split ratio of 0.8 but use different random seeds to get different training datasets, i.e., the data sequence has been shuffled. The averaged RMSE ($\overline{\text{RMSE}}$) is then used as an error metric.

Group	Features	Algorithms	RMSE (eV)			
			1st	2nd	3rd	$\overline{\text{RMSE}}$
1	T_m	GLM, SE, GBM	0.7340	0.6603	0.7482	0.7142
2	N, T_m	SE	0.6970	0.6671	0.7493	0.7045
3	R_A, R_B, T_m	SE	0.6967	0.6145	0.6529	0.6547
4	m_A, m_B, T_m	SE	0.7150	0.5959	0.6904	0.6671
5	χ_A, χ_B, T_m	GBM, SE	0.6857	0.6257	0.6623	0.6579
6	N, R_A, R_B, T_m	SE	0.6543	0.6003	0.6696	0.6414
7	N, m_A, m_B, T_m	GBM, SE	0.6507	0.5912	0.6786	0.6402
8	N, χ_A, χ_B, T_m	GBM, SE	0.6265	0.6525	0.7271	0.6687
9	$N, R_A, R_B, m_A, m_B, T_m$	SE	0.6283	0.5891	0.6646	0.6273
10	$N, R_A, R_B, \chi_A, \chi_B, T_m$	SE, GBM	0.6291	0.5919	0.6198	0.6136
11	$N, m_A, m_B, \chi_A, \chi_B, T_m$	GBM, SE, XGBoost	0.6107	0.5790	0.6682	0.6193
12	$R_A, R_B, m_A, m_B, \chi_A, \chi_B, T_m$	GBM, SE	0.6789	0.5831	0.6138	0.6253
13	$N, R_A, R_B, m_A, m_B, \chi_A, \chi_B$	GBM, SE	0.9414	0.8892	0.8991	0.9099
14	$N, R_A, R_B, m_A, m_B, \chi_A, \chi_B, T_m$	GBM, SE	0.5907	0.5752	0.6139	0.5933

*GLM: Generalized Linear Models, **SE:** Stacked Ensemble, **GBM:** Gradient Boosting, **XGBoost:** Extreme Gradient Boosting.

Supplementary Table 7. Comparison of the ML predicted sublimation enthalpies in (eV) with thermodynamic FactSage database and DFT calculation, MAE_w, $RMSE_w$ and R_w^2 are the results without Cr, XG in the table is XGboost.

Methods	Sr	Ni	Cu	Cr	NaCl	NaF	SiO_2	ZrO_2	MAE	RMSE	R^2	MAE_w	$RMSE_w$	R_w^2
**FactSage**	1.6997	4.4567	3.5031	3.7857	2.0044	2.4841	5.6377	7.5099	-	-	-	-	-	-
**DFT**	1.6122	4.7700	3.4863	4.0001	2.1130	2.5304	5.7650	6.8977	0.1908	0.2639	0.9793	0.1874	0.2702	0.9810
**XG-6D**	1.9802	4.2665	3.3383	4.8734	2.1316	2.7558	5.4931	6.4944	0.4103	0.5553	0.9085	0.3135	0.4283	0.9524
**XG-7D**	2.1559	4.0490	3.4531	4.8678	2.3257	2.4684	5.6126	6.4872	0.4226	0.5807	0.9000	0.3284	0.4670	0.9434
**XG-8D**	2.1374	4.0783	3.4331	4.8977	2.1550	2.4994	5.6381	7.1231	0.3189	0.4675	0.9352	0.2056	0.2705	0.9810
**SVR-6D**	1.8215	3.6758	3.0445	4.7670	2.1257	2.6091	5.2114	6.5729	0.4940	0.6008	0.8929	0.4244	0.5244	0.9286
**SVR-7D**	1.8521	4.0246	3.4193	4.8441	2.3352	2.8272	5.1254	6.6126	0.4763	0.5736	0.9024	0.3931	0.4647	0.9439
**SVR-8D**	1.8876	4.0745	3.4206	4.8001	2.1055	2.6888	5.5735	6.5027	0.3805	0.5348	0.9152	0.2900	0.4241	0.9533
**DTR-6D**	2.3261	4.1864	3.7313	4.8759	2.1665	2.7672	4.8759	7.2141	0.4647	0.5568	0.9080	0.3754	0.4296	0.9521
**DTR-7D**	2.3261	4.0130	3.4265	4.8759	2.5038	2.5038	4.8759	7.2141	0.4767	0.5811	0.8998	0.3891	0.4649	0.9439
**DTR-8D**	2.3261	4.0130	3.4265	4.8759	2.5038	2.5038	4.8759	7.2141	0.4767	0.5811	0.8998	0.3891	0.4649	0.9439
**GPR-6D**	2.1259	4.3357	3.5014	5.1889	2.3248	2.5265	4.8761	6.2551	0.5414	0.7437	0.8360	0.4183	0.5922	0.9089
**GPR-7D**	2.3373	4.2368	3.4691	4.9294	2.3622	2.5550	4.9027	6.1180	0.5738	0.7395	0.8378	0.4924	0.6619	0.8862
**GPR-8D**	2.3378	4.1907	3.4558	4.8794	2.3489	2.5293	4.9637	6.1698	0.5561	0.7113	0.8499	0.4793	0.6382	0.8942

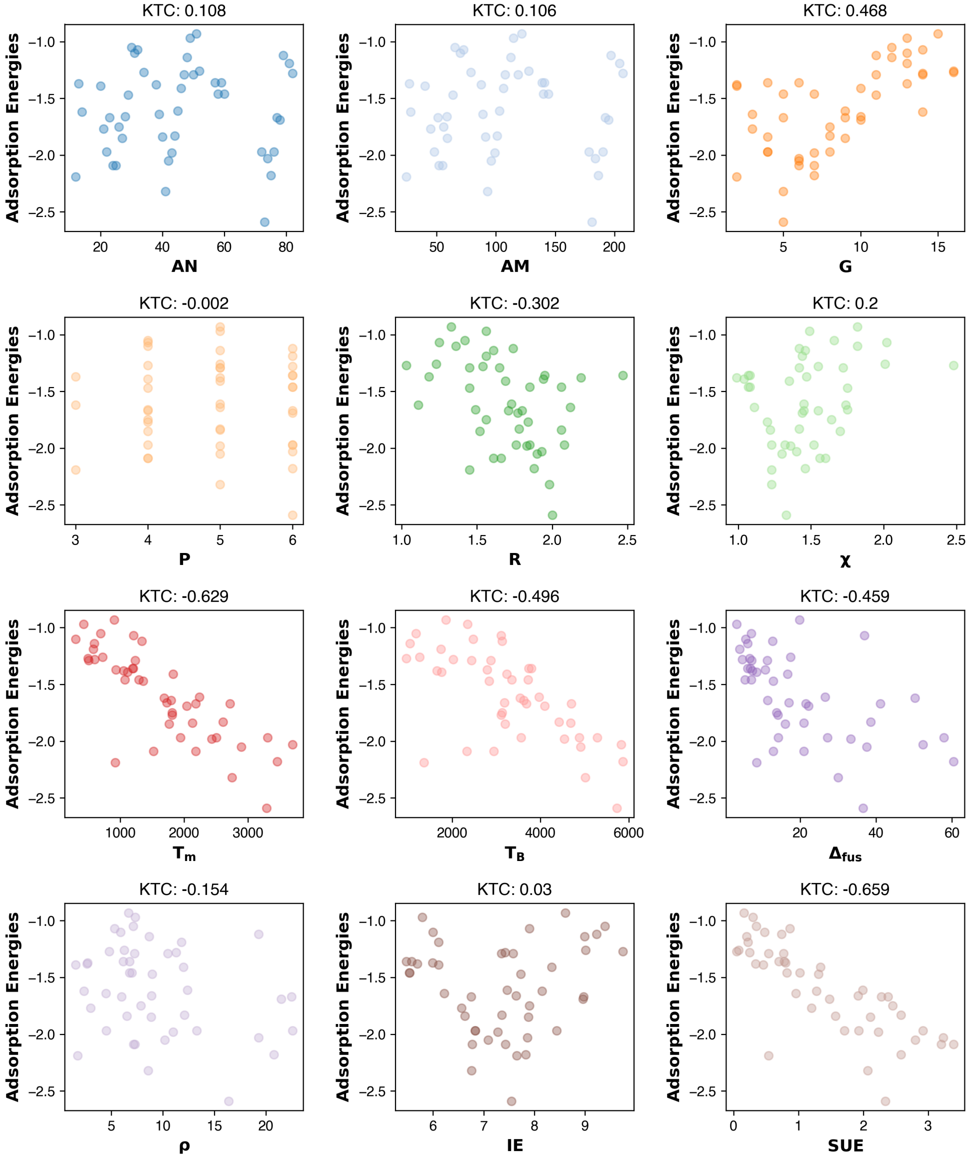

Supplementary Figure 1. Univariate correlation analysis for adsorption energies dataset.

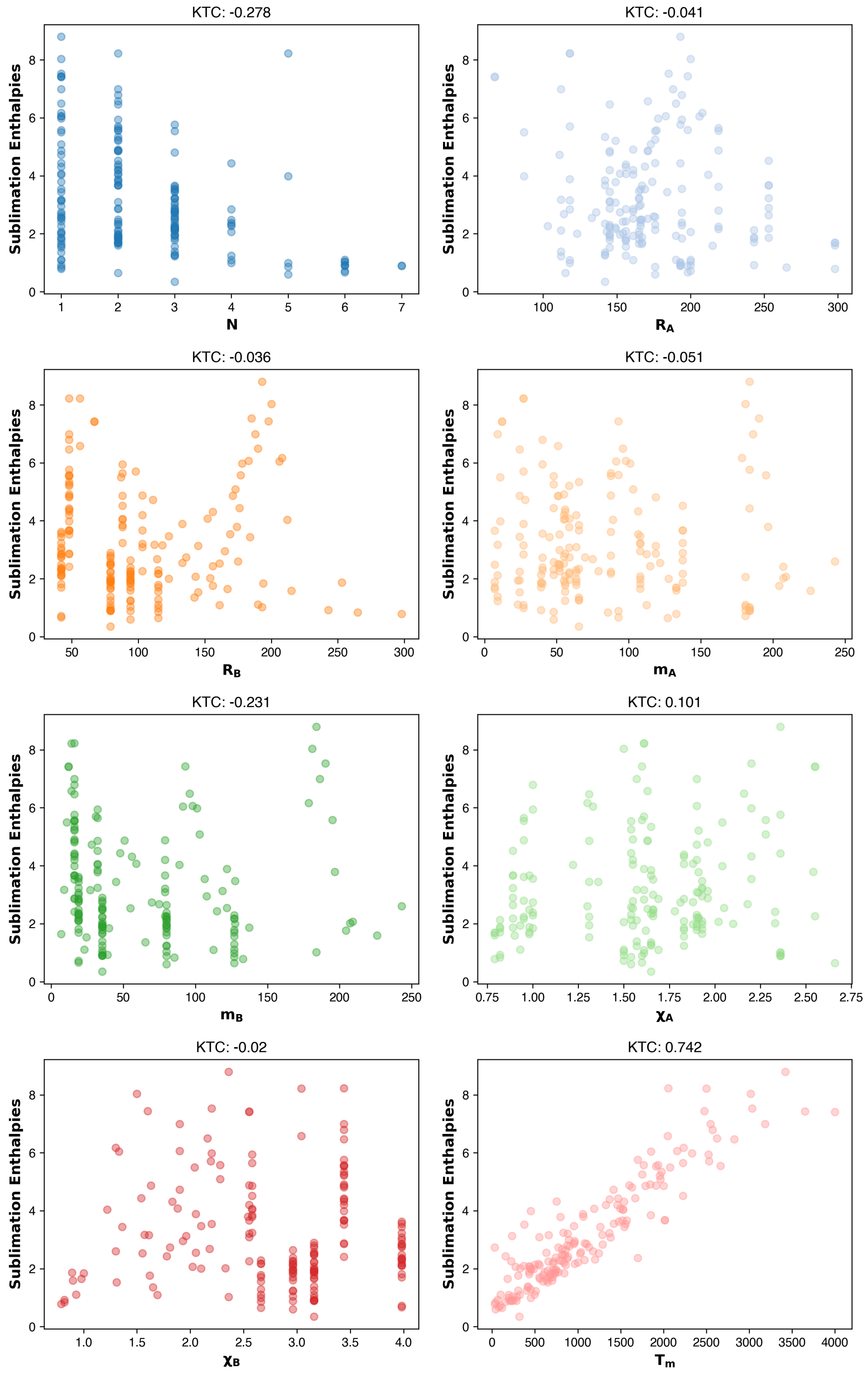

Supplementary Figure 2. Univariate correlation analysis for sublimation enthalpies dataset.

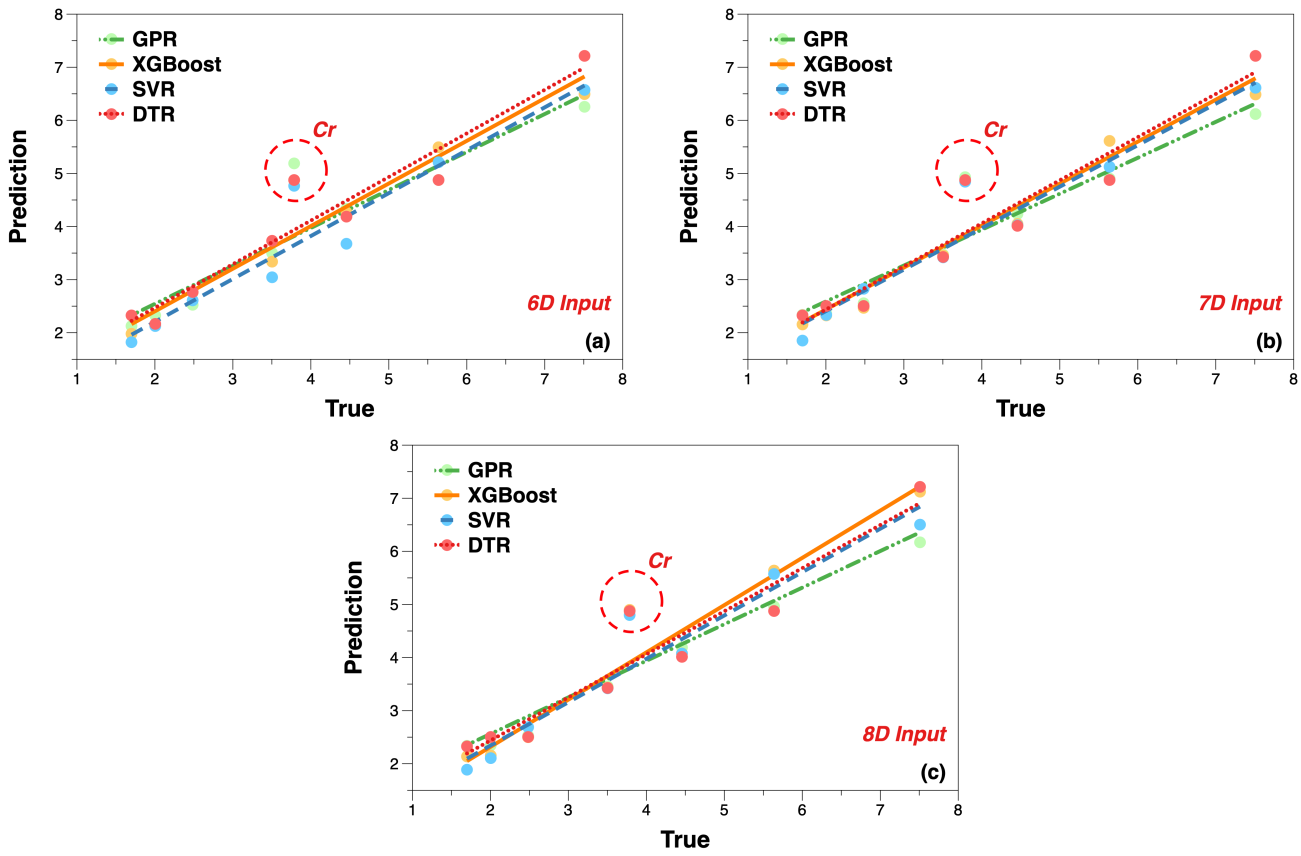

Supplementary Figure 3. Plot of the predicted sublimation enthalpies versus the true values, (a) 6D input, (b) 7D input and (c) 8D input.

References

1. Toyao, T. *et al.* Toward effective utilization of methane: machine learning prediction of adsorption energies on metal alloys. *The J. Phys. Chem. C* **122**, 8315–8326 (2018).
